# In the Blood

**Published:** 1879-10

**Authors:** 


### EDITORIAL, ETC.

*In the Blood.*—It is now some five years since Prof. Carl Heitzman announced his theory of what he deemed was an important discovery in respect to the "histology of protoplasm," claiming that this substance, no matter where found or however placed, invariably contained a net-work of threads and granules inclosing a fluid, the threads and granules being the living matter. Although not recognized in this country, Prof. H. asserts that this doctrine is accepted by more than a dozen of the best microscopists of England and the Continent. Of late he has come forward with an application of his theory, which, if substantiated, must have a controlling effect on differential diagnosis in the near future. Boldly he claims that a drop of blood or urine, under the microscope, will enable the microscopist to tell not only with what disease the patient is suffering, but will also enable the observer to determine whether the incipient disease is to be of slow or acute type—in a word, that the blood or urine will tell the secrets of a patient's constitution.

If it be true, as is claimed, that the pus-corpuscles of a healthy and strong person contain a greater amount of living matter than those of a person enfeebled by disease, and that the colorless corpuscles of the blood in health contain a large amount of these protoplasmic granules, and consequently an enfeebled person only a few, it would be a step largely forward. Whether its practical bearing will be as valuable as is predicted is a question. The best microscopists differ and wrangle. The work itself is a life-long one, and the probability seems that the mass of practicing physicians would not be at all able to utilize these discoveries, even were their authority acknowledged beyond dispute. Still, all such advances are eminently satisfactory, if only as establishing sound pathological theories; and if they do no other good, will at least tend to diminish irrational drugging.

It is worthy of mention that Prof. Heitzman in a discussion before the New York Odontological Society, in December last, announced as a theory that "inflamation in its first or earliest stage—its mildest degree—consists in a reduction of tissue to its medullary condition. H.

